# Identification, Isolation, and Characterization of Medipeptins, Antimicrobial Peptides From *Pseudomonas mediterranea* EDOX

**DOI:** 10.3389/fmicb.2021.732771

**Published:** 2021-09-14

**Authors:** Lu Zhou, Anne de Jong, Yunhai Yi, Oscar P. Kuipers

**Affiliations:** Department of Molecular Genetics, University of Groningen, Groningen, Netherlands

**Keywords:** *Pseudomonas*, genome mining, biosynthesis, cyclic lipopeptides, mode of action

## Abstract

The plant microbiome is a vastly underutilized resource for identifying new genes and bioactive compounds. Here, we used *Pseudomonas* sp. EDOX, isolated from the leaf endosphere of a tomato plant grown on a small farm in the Netherlands. To get more insight into its biosynthetic potential, the genome of *Pseudomonas* sp. EDOX was sequenced and subjected to bioinformatic analyses. The genome sequencing analysis identified strain EDOX as a member of the *Pseudomonas mediterranea*. *In silico* analysis for secondary metabolites identified a total of five non-ribosomally synthesized peptides synthetase (NRPS) gene clusters, related to the biosynthesis of syringomycin, syringopeptin, anikasin, crochelin A, and fragin. Subsequently, we purified and characterized several cyclic lipopeptides (CLPs) produced by NRPS, including some of the already known ones, which have biological activity against several plant and human pathogens. Most notably, mass spectrometric analysis led to the discovery of two yet unknown CLPs, designated medipeptins, consisting of a 22 amino acid peptide moiety with varying degrees of activity against Gram-positive and Gram-negative pathogens. Furthermore, we investigated the mode of action of medipeptin A. The results show that medipeptin A acts as a bactericidal antibiotic against Gram-positive pathogens, but as a bacteriostatic antibiotic against Gram-negative pathogens. Medipeptin A exerts its potent antimicrobial activity against Gram-positive bacteria *via* binding to both lipoteichoic acid (LTA) and lipid II as well as by forming pores in membranes. Collectively, our study provides important insights into the biosynthesis and mode of action of these novel medipeptins from *P. mediterranea* EDOX.

## Introduction

*Pseudomonas* bacteria are found in abundance in nature and have a fascinating metabolic diversity, which is linked to their ability to colonize a diverse range of ecological niches (Gotze and Stallforth, 2020). *Pseudomonas* species have a wide metabolic range and can produce diverse metabolites, including cyclic lipopeptides (CLPs), 2,4-diacetylphloroglucinol (DAPG), pyrrolnitrin (PRN), pyoluteorin (PLT), phenazines (PHZs), 2,5-dialkylresorcinol, gluconic acid, quinolones, rhamnolipids, siderophores, and hydrogen cyanide. It is obvious that the bacteria from the genus *Pseudomonas* represent a rich source of natural products (Gross and Loper, 2009; D’aes et al., 2010; Masschelein et al., 2017; Zhao et al., 2019).

With the progress made by up-to-date nucleotide-sequencing techniques, a new age of natural products discovery has begun, greatly accelerated by the pace of genome sequencing of microorganisms. This advancement has paved the way for the effective prediction of gene clusters encoding new bioactive compounds and the understanding of the underlying biosynthetic processes (Vater et al., 2018). Genome mining studies of *Pseudomonas* species have shown a wide variation of biosynthetic gene clusters (BGCs) between genera, species, and even strains within the same species (Loper et al., 2012; Van Der Voort et al., 2015). *Pseudomonas* species produce non-ribosomally synthesized peptides (NRPs), which are oligopeptides (8–25 amino acids) that are N-terminally acylated with a fatty acid. This kind of bacterial natural product is formed by non-ribosomally synthesized peptide synthetases (NRPSs), which may insert non-canonical amino acids into extending peptides (Schwarzer et al., 2003; Weissman, 2015; Gotze and Stallforth, 2020). The vast majority of *pseudomonal* lipopeptides are not linear, but rather have a macrolactone ring shaped between the peptide’s C-terminus and the hydroxyl group of a serine or threonine. The macrocycles vary in size from four to 10 amino acids (Gotze and Stallforth, 2020).

All known CLPs produced by *Pseudomonas* species can be classified into 14 major groups, according to the length and structure of the oligopeptide. The majority of them have antifungal and antibacterial properties, while others have antioomycete, antiviral, antiprotozoan, and antitumor properties (Geudens and Martins, 2018). CLPs inhibit bacterial growth by affecting either cell wall biosynthesis or cell membrane integrity. In brief, CLPs can insert into plasma membranes and alter their microdomain membrane fluidity. This can result in a rearrangement of plasma membrane architecture, which can cause depolarization and ion leakage or influx, both of which can cause cell death or compromised proliferation (Muller et al., 2016). In general, most CLPs from *Pseudomonas* species tested exhibit activity against Gram-positive bacteria in the low micromolar range, but not against Gram-negative bacteria (Raaijmakers et al., 2006; Reybroeck et al., 2014).

## Materials and Methods

### Isolation of Bacteria

The isolation of bacteria from leaves was conducted according to a previous protocol (Zhou et al., 2021). In brief, 1g tomato leaves was surface-sterilized for 1min. in 70% ethanol and 3min. in 0.5% NAClO solution combined with one droplet Tween 80 per 100ml solution. After that, leaves were rinsed 5 times in sterilized deionized water. Subsequently, the leaves were macerated in 9ml of 10mM MgSO_4_ buffer with a mortar to get the tissue suspension. After dilution 10^3^–10^6^ times with 10mM MgSO_4_ buffer, the suspension was spread on lysogeny broth (LB) agar plates. To obtain single colonies, the plates were incubated at 28°C for 24–48h. Surface sterilization was validated by spreading aliquots of the final rinsing by deionized water on LB agar plates (if no organism growth was detected after 7days, surface sterilization was deemed successful).

### Whole Genome Sequencing and Assembly

Genomic DNA was extracted from a culture grown from a single colony using a GenElute bacterial genomic DNA kit (Sigma) according to the manufacturer’s instructions. The genomes were sequenced at GATC Biotech (Germany) using an Illumina HiSeq sequencing system. Raw reads were validated by FastQC version 0.11.5 (Andrews, 2010). Trimmomatic version 0.38 was used to delete low-quality reads (Bolger et al., 2014). Subsequently, the reads were assembled *de novo* using SPAdes version 3.11.1 (Bankevich et al., 2012). Default parameters were used for all software unless specified. The draft genomes were then annotated by the Rapid Annotations using Subsystems Technology (RAST) server. The dDDH and ANI values were determined using the online applications TYGS (Meier-Kolthoff and Goker, 2019) and JSpeciesWS (Richter et al., 2016), respectively, for the categorization of species affiliations.

### Genome Mining of Biosynthetic Gene Clusters

In order to identify biosynthesis gene clusters (BGCs) in strain EDOX, the draft genome sequence was constructed into a pseudomolecule using the Medusa web server^1^ (Bosi et al., 2015) with multiple closely related strains as references. The pseudomolecules were then sent to antiSMASH 5.0 (Blin et al., 2019) and BAGEL 4 (van Heel et al., 2018) for BGC mining.

### Purification of Antimicrobial Compounds From the EDOX Strain

For antimicrobial compounds purification, seed culture of strain EDOX was cultivated in 4ml King’s B (KB) media contained in a glass tube and shaken on a rotary shaker for 24h at 28°C, 210rpm. Subsequently, the seed culture was inoculated in 2-L flasks containing 400ml KB media at 210rpm for 48h. The supernatant was collected by centrifugation (10,000×*g*, 30min) and acidified to pH 2 with 6N hydrochloric acid (HCl) and kept overnight at 4°C for precipitation of CLPs. After that, the precipitates were collected by centrifugation (10,000×*g*, 30min) and extracted using methanol. To get crude CLPs extracts, methanol was evaporated at room temperature. The crude extracts were dissolved in Milli-Q water and filtered with 0.45μm Durapore™ membrane and then subjected to a reverse high-performance liquid chromatography (HPLC) equipped with Aeris widepore 3.6μ XB-C18 250×4.6mm column for purification. The mobile phases were HPLC-grade water supplemented with 0.1% trifluoroacetic acid (TFA; solvent A) and acetonitrile supplemented with 0.1% TFA (solvent B). An elution gradient of solvent A/solvent B (75:25 to 0:100) was applied at a flow rate of 1ml/min, for compound purification. A UV detector set at a wavelength of 214nm was used to monitor the compound’s elution.

### Liquid Chromatography–Tandem Mass Spectrometry Analysis

A Q-Exactive Orbitrap™-based mass spectrometer equipped with an Ultimate 3000 UPLC, an ACQUITY BEH C18 column (2.1 50mm, 1.7m particle scale, 200; Waters), a HESI-II ion source, and an Orbitrap detector was used to perform LC–MS/MS to obtain insight into the molecular configurations of the peptides. In each run, a 30-min gradient of 5–90% MeCN/Milli-Q water (v/v) with 0.1% formic acid (v/v) was used with a flow rate of 0.35ml/min. The doubly and triply charged ions of the compound of interest were selected by MS/MS in a separate run in PRM mode.

### Determination of the Minimum Inhibitory Concentration

MIC tests were conducted in triplicate using liquid growth inhibition microdilution assays in sterile polypropylene microtiter plates according to the Clinical and Laboratory Standards Institute (CLSI) guideline (Wiegand et al., 2008; Hindler and Richter, 2016). The pathogenic indicator strains were streaked on LB agar plates first, and then, single colonies were selected and incubated in LB broth overnight. The final bacteria inoculum was prepared in CAMHB (cationic adjusted Mueller-Hinton broth, Sigma-Aldrich) at 5×10^5^ CFU/ml. The antimicrobials were serially diluted two times, and 50μl of diluted bacterial suspension was applied to each well to make the final volume 100μl. Without stirring, the microtiter plates were incubated for 48h at 30°C. The OD_600_ was measured using a microplate reader (Tecan Infinity F200) to determine growth inhibition. The MIC value is described as the lowest concentration of antimicrobials that inhibits a pathogen’s visible growth.

### Time-Kill Assay

This assay was performed according to a previously described protocol with small modification (Ling et al., 2015). An overnight culture of either *Staphylococcus aureus* 533R4 or *X. translucens* pv. graminis LMG587 was diluted 50-fold in MHB and incubated at 30°C with the shaker at 220rpm. Bacteria were grown to an OD_600_ of 0.5, and then, the concentration of cells was adjusted to ≈5×10^5^ cells per ml for *X. translucens* and ≈2×10^8^ for *S. aureus*. Bacterial cultures were then exposed to 10X MIC antimicrobials at 30°C and 220rpm. Bacteria not treated with medipeptin A were used as a negative control. Bacteria treated with vancomycin or colistin were used as a positive control. At desired time points (0, 1, 2, 3, 4, 5, and 6h), 10μl culture was taken and tenfold serially diluted. Subsequently, each sample was plated on MHA plates. After incubation at 30°C for 48h, colonies were counted and the number of CFU per ml was calculated. Each experiment was performed in triplicate.

### Membrane Permeabilization Assays

In order to investigate whether medipeptin A could permeabilize the cell membranes, a commercial LIVE/DEAD Baclight Bacterial Viability Kit (Invitrogen) was used. Cells of *S. aureus* were grown in LB overnight and diluted to an OD_600_ of 0.2. Compound medipeptin A was added at a concentration of 40μg/ml (5X MIC) in 1ml culture, while nisin was added at a concentration of 2.5μg/ml. The same amount of DMSO was added to the control. Cells were treated at room temperature for 1h before harvest. The harvested cells were washed and resuspended in a 200μl 0.85% saline (NaCl) solution. Two different dyes (3.34mM SYTO®9 and 20mM propidium iodide) were added at a ratio of 1:1 (v/v). Cells were stained in the dark for 30min, and a 2μl sample was mounted on a 1% agarose pad before being imaged using a DeltaVision Elite microscope.

### Lipoteichoic Acid Binding Assay

Cultures of *S. aureus* grown overnight were diluted to an OD_600_ of 0.05 in a 96-well plate and incubated in a microplate spectrophotometer (1,000rpm at 30°C). When the OD_600_ reached 0.1, medipeptin A (dissolved in DMSO) was added to each well at a final concentration of 24μg/ml (3X MIC), and the same volume of DMSO was used as a negative control. Nisin (at a final concentration of 2μg/ml) was used as a positive control for *S. aureus*. Lipoteichoic acid (LTA; at a final concentration of 100μg/ml) was added to look into the binding activity of medipeptin A. The growth curve was recorded using the microplate spectrophotometer under the same circumstances for the next 16h, with four replicates for each treatment. LTA is negatively charged and is thought to influence Gram-positive bacteria’s sensitivity to cationic antimicrobial peptides like defensin and nisin *via* electrostatic contact (Yang et al., 2017). The LTA added in each sample could compete with LTA on cell envelopes for binding with cationic antimicrobial peptides, which could cause the reduction in antimicrobial activity of peptides. Comparing with only peptides treatment, medipeptin A binding to LTA is indicated by the restoration of cell growth under treatment of LTA and peptides together.

### Lipid II Binding Assay

An overnight culture of *S. aureus* was mixed with precooled LB agar (around 55°C) at a final concentration of 0.1% (v/v), and then, the mixed media was poured onto Petri dishes to obtain pathogen-fusion agar plates. Binding of medipeptin A and lipid II was further evaluated by spotting of purified lipid II (300μM, 4μl) to the edge of the halo caused by each compound. Briefly, medipeptin A or vancomycin was exposed to 100°C for 30min. Then, medipeptin A or vancomycin (with or without heat treatment) was loaded to the agar plate, and after the solution drops had dried, purified lipid II was spotted to the edge (identified by a pre-experiment) of the inhibition halo formed by antibiotics. After the lipid II solution drops had dried, the plates were transferred to a 30°C incubator for overnight incubation. The decrease in halo size indicates the binding of lipid II and medipeptin A.

## Results

### Isolation and Identification of Strain EDOX

The strain *Pseudomonas* sp. EDOX, isolated from the healthy tomato leave endosphere, showed antagonistic activity against both bacterial and fungal pathogens in our preliminary research. The genomic DNA was isolated and sequenced. The draft genome consists of a 6,142,102bp. A total of 5,335 CDSs and 66 RNA genes including rRNA, tRNA, and ncRNA genes were identified. The general features of the genome of strain EDOX are summarized in Table 1. To classify at the species level, ANI and dDDH values were determined. Strain EDOX exhibited ≥99.27% ANI and ≥96.80% dDDH compared with the closest related species, that is, *Pseudomonas mediterranea* CFBP 5447, which is above the gold standard threshold value of 95% ANI and 70% dDDH for the delineation of species. Therefore, strain EDOX was identified as *Pseudomonas mediterranea* EDOX. This whole genome analysis result has been deposited at DDBJ/ENA/GenBank under the accession number GCA_016735085.1.

**Table 1:** Draft genome features of *P. mediterranea* EDOX.

Characteristics	Genome
Size (bp)	6,142,102
G+C content (%)	61.4
N50 (bp)	422,748
Genes number	5,401
Contigs	83
CDS number	5,335
rRNA	4
tRNA	58
ncRNA	4

### Automated Searching for Secondary Metabolite Clusters Using the antiSMASH Pipeline

By bioinformatic evaluation of the genome sequence of EDOX using antiSMASH 6.0, 12 biosynthetic gene clusters were identified (Table 2). Five of these are NRPs BGCs predicted to be related to the biosynthesis of syringomycin (Scholz-Schroeder et al., 2003), syringopeptin (Scholz-Schroeder et al., 2001), anikasin (Götze et al., 2017), crochelin A (Baars et al., 2018), and fragin (Jenul et al., 2018). Two were identified as type I PKs-NRPs hybrid BGCs and related to the biosynthesis of lankacidin C (Mochizuki et al., 2003) and entolysin (Vallet-Gely et al., 2010). One siderophore BGC and One Ripp-like BGC were also found in the genome. In addition, four other BGCs were discovered. Type “other BGC” is a cluster containing a secondary metabolite-related protein that does not fit into any other category.

**Table 2:** Secondary metabolite and antibiotic gene clusters identified from *P. mediterranea* EDOX using antiSMASH 6.0 and BAGEL4.

BGCs cluster	Type	Most similar known BGCs	MIBiG BGC ID	Mass spectrometry Identification (supernatant of EDOX)
1	Type I PKs-NRPs hybrid	Lankacidin C (13% of genes show similarity)	BGC0001100	not detected
2	NRPs	Syringomycin (100% of genes show similarity)	BGC0000437	Cormycins (Strano et al., 2015)
3	NRPs	Syringopeptin (100% of genes show similarity)	BGC0000438	edox-1, edox-2^*^
4	Siderophore	-	-	not detected
5	NRPs	Anikasin (22% of genes show similarity)	BGC0001509	not detected
6	Type I PKs-NRPs hybrid	Entolysin (17% of genes show similarity)	BGC0000344	not detected
7	NRPs	Crochelin A (15% of genes show similarity)	BGC0002001	not detected
8	Other	Fengycin (13% of genes show similarity)	BGC0001095	not detected
9	Other	-	-	not detected
10	RiPP	-	-	not detected
11	Other	APE Vf (45% of genes show similarity)	BGC0000837	not detected
12	NRPs	Fragin (37% of genes show similarity)	BGC0001599	not detected

Hyphens (−) were indicating that no similarity was found with known biosynthetic gene clusters (BGCs) in MIBiG repository.

* edox-1 and edox-2 were later renamed as medipeptin A and medipeptin B.

### Purification and Identification of Lipopeptides

**Figure 1:**
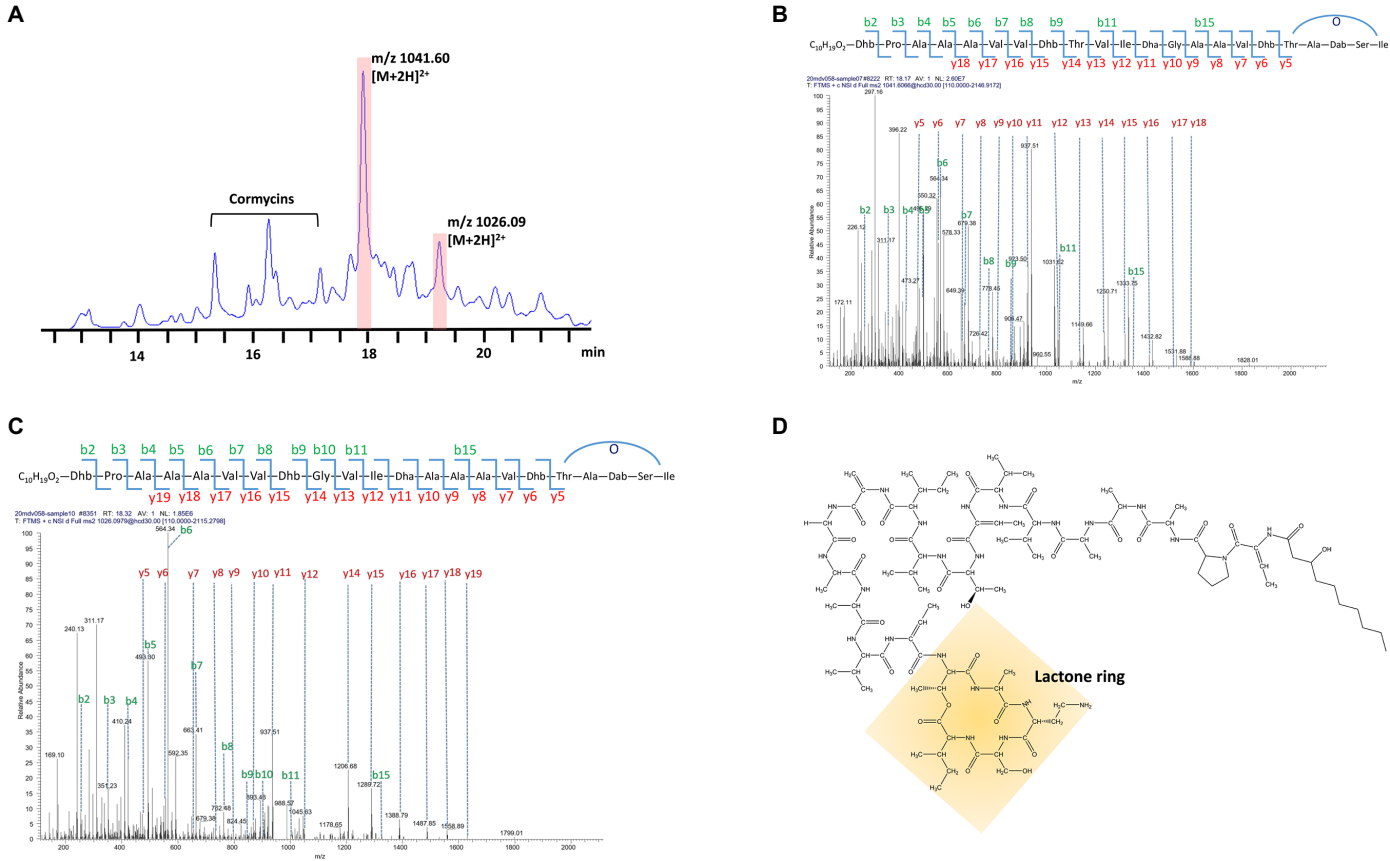
Characterization of Purified CLPs produced by strain EDOX. **(A)** HPLC chromatographic profile of crude extract from supernatant of strain EDOX. **(B)** *De novo* sequencing of medipeptin A (m/z 2082.20) predicted from gene cluster 3 obtained by genome mining of *P. mediterranea* EDX by using LC–MS/MS. **(C)** *De novo* sequencing of the medipeptin B (m/z 2051.18) encoded by gene cluster 3 by using LC–MS/MS. **(D)** The proposed chemical structure of medipeptin A.

**Figure 2:**
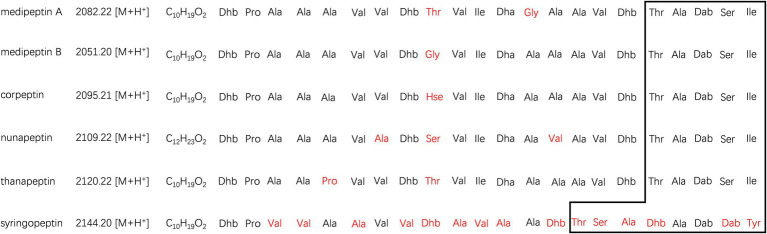
Comparison of medipeptins with similar CLPs. The macrolactone ring shaped between the C-terminus of each peptide and a hydroxyl group of a threonine is remarked by square area. The different parts in medipeptins, compared with other CLPs, were marked in red. Non-canonical amino acids are abbreviated as follows: Dhb, dehydrobutyrine; Hse, homoserine; Dha, dehydroalanine; and Dab, 2,4-diaminobutyric acid.

**Figure 3:**
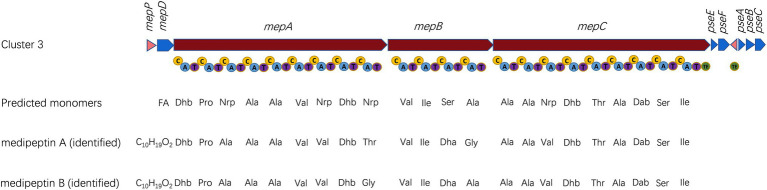
Characterization of biosynthetic gene cluster (BGC) of medipeptins. Transporter genes are indicated in blue; core biosynthetic genes are shown in red; and regulator genes are labeled in pink. Three core biosynthetic genes (*mepA-C*) contain 22 modules including adenylation (A), condensation (C), thiolation (T) and thioesterase (TE) domains. The predicted amino acids sequences are compared with identified medipeptins below. FA, fatty acid; and Nrp, uncertain amino acid.

Two compounds, initially named edox-1 and edox-2, were isolated and purified from strain EDOX (Table 2). The compounds were subjected to LC–MS and LC–MS/MS, which showed m/z values of 1041.60 [M+2H]^2+^ (Figure 1B) and 1026.09 [M+2H]^2+^ (Figure 1C), respectively. As shown in Figure 1, these two lipopeptides were identified as 22 amino acid cyclic lipopeptides, that is, Dhb-Pro-Ala-Ala-Ala-Val-Val-Dhb-Thr-Val-Ile-Dha-Gly-Ala-Ala-Val-Dhb-Thr-Ala-Dab-Ser-Ile and Dhb-Pro-Ala-Ala-Ala-Val-Val-Dhb-Gly-Val-Ile-Dha-Ala-Ala-Ala-Val-Dhb-Thr-Ala-Dab-Ser-Ile, respectively. Analyses using high-resolution LC–MS/MS, the primary ions at m/z 2082.20 [M+H]^+^ and 2051.18 [M+H]^+^ showed a distinct sequence tag for 16 amino acid residues with the sequences Pro-Ala-Ala-Ala-Val-Val-Dhb-Thr-Val-Ile-Dha-Gly-Ala-Ala-Val-Dhb and Dhb-Pro-Ala-Ala-Ala-Val-Val-Dhb-Gly-Val-Ile-Dha-Ala-Ala-Ala-Val-Dhb, respectively. Furthermore, the mass spectrum showed two larger fragments of m/z 254.167 and m/z 473.264. The m/z 254.167 fragment’s molecular formula corresponded to a Dhb residue linked to 3-OH decanoic acid also known as monomer C10:0-OH(3), which is similar to the structure of corpeptin (Emanuele et al., 1998). The m/z 473.264 fragment’s molecular formula has a Thr-Ala-Dab-Ser-Ile cyclic tail, which was consistent with nunapeptin (Michelsen et al., 2015; Hennessy et al., 2017) and corpeptin. Lipopeptides edox-1 and edox-2 showed some structural resemblance to nunapeptin from *P. fluorescens* In5 (Michelsen et al., 2015), to braspeptin from *Pseudomonas* sp. 11K1 (Zhao et al., 2019), and to corpeptin A from *P. corrugate* CFBP 5454 (Emanuele et al., 1998; Strano et al., 2015) as well as to thanapeptin from *Pseudomonas* sp. SH-C52 (Van Der Voort et al., 2015; Figure 2). Therefore, in agreement with the naming of corpeptin produced by *P. corrugate*, we designated the edox-1 and edox-2 as medipeptin A and medipeptin B, respectively. Based on the genome mining data, these two lipopeptides purified from the bacteria culture are obviously in accordance with cluster 3 identified by antiSMASH 6.0 (Figure 3).

### Biosynthesis of Medipeptins by *Pseudomonas* sp. EDOX

Based on the genome mining, a total of 12 BGCs were discovered in the genome of strain EDOX. We identified and characterized the gene cluster for medipeptin biosynthesis (Figure 3). This BGC contains 11 genes, including three large biosynthetic genes (*mepA-C*), six genes associated with export of peptides (*mepD*, *pseA-C*, *pseE*, and *pseF*), one gene related to biosynthesis regulation of medipeptins (*mepP*), and one gene encoding a thioesterase (TE) domain. Based on the structure of medipeptins, we determined that medipeptin biosynthesis is catalyzed by genes *mepA-C* in a colinear manner, which is identical to the biosynthesis of corpeptin (Strano et al., 2015). Moreover, medipeptin A and B show two amino acid residues difference in corpeptin and nunapeptin, one amino acid residue difference in thanapeptin, and 16 amino acids residues difference in syringopeptin (Figure 2). All these differences may lead to distinctive antimicrobial activity of each lipopeptide.

### Determination of MIC

The antimicrobial activity of medipeptin A against pathogenic bacteria was determined by MIC assays. In our study, purified medipeptin A showed potent antimicrobial activity against several tested Gram-negative pathogens, including *X. translucens*, *P. syringae*, and *X. campestris*, with a MIC value of 4–16μg/ml (Table 3). In addition, purified medipeptin A also showed potent antimicrobial activity against Gram-positive bacteria, including *M. flavus*, *S. aureus*, and *B. cereus*, with a MIC value of 2–8μg/ml (Table 3).

**Table 3:** MIC value of medipeptin A against pathogenic bacteria.

Type of pathogens	Pathogens	MIC (μg/ml)		
		medipeptin A	Colistin	Vancomycin
Gram-negative	*Xanthomonas campestris* pv. *campestris* NCCB92058	16	1	nd
	*Xanthomonas translucens* pv. *graminis* LMG587	4	1	nd
	*Pseudomonas syringae* pv. *tomato* DC3000	8	1	nd
	*Ralstonia syzygii* subsp. *Syzygii* LMG6969	>32	1	nd
	*Escherichia coli* ET8	>32	2	nd
	*Klebsiella pneumoniae* LMG20218	>32	2	nd
	*Acinetobacter baumannii* LMG01041	>32	2	nd
Gram-positive	*Staphylococcus aureus* subsp. *aureus* 5334R4	8	nd	0.5
	*Bacillus cereus* ATCC14579	8	nd	0.5
	*Micrococcus flavus* (not pathogenic)	2	nd	0.25

nd was indicating that MIC values were not detected.

### Medipeptin A Acts as a Bactericidal Antibiotic Against Gram-Positive Pathogens but as a Bacteriostatic Antibiotic Against Gram-Negative Pathogens

**Figure 4:**
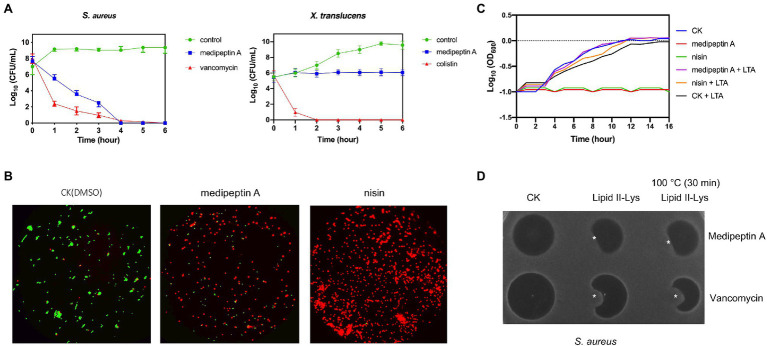
Investigation of the mode of action of medipeptin A. **(A)** Time killing curve of medipeptin A (10X MIC) to *S. aureus* and X. translucens pv. graminis. **(B)** Membrane permeability of medipeptin A (5X MIC) to *S. aureus*. Nisin was added at a concentration of 2.5μg/ml, and the same amount of DMSO was treated as control. Cells with a compromised membrane integrity were stained in red, while cells with intact membrane were stained in green. **(C)** Medipeptin A binds to LTA. Medipeptin A (24μg/ml 3X MIC) was added to each well, and the same volume of DMSO was used as a negative control. Nisin (2μg/ml) was used as a positive control. **(D)** Medipeptin A binds to lipid II (Gram-positive lipid II, lipid II-Lys). Medipeptin A was added at a concentration of 200μg/ml; vancomycin was added at a concentration of 200μg/ml; ^*^the position of lipid II-Lys was added at a concentration of 300μM, 4μl.

The time dependency of antibiotic action is a sound method for determining whether a compound is bacteriostatic or bactericidal. In this study, we monitored the killing kinetics of medipeptin A against *X. translucens* and *S. aureus*. Lipopeptide medipeptin A showed bactericidal capacity against *S. aureus*, and it killed all the *S. aureus* in 4h at a desirable concentration (10X MIC; Figure 4A). However, the growth of *X. translucens* cells treated with medipeptin A just stopped or showed limited growth when compared to the untreated cells (Figure 4A). These results demonstrate that medipeptin A may act as a bactericidal antibiotic against *S. aureus* and acts as a bacteriostic antibiotic against *X. translucens*.

### Medipeptin A Permeabilizes the Cell Membrane of Gram-Positive Bacteria

We analyzed whether medipeptin A can permeabilize the cell membrane of *S. aureus* using a commercial LIVE/DEAD Baclight Bacterial Viability Kit (Invitrogen). Cells with an intact membrane will stain green, whereas cells with a compromised membrane will stain red. The results show that medipeptin A disrupts the membrane of *S. aureus* cells when compared to the control treated with DMSO, which showed no influence on the membrane permeability of *S. aureus* cells (Figure 4B). These results demonstrate that medipeptin A acts as a potent antibiotic against Gram-positive bacteria by disrupting the cytoplasmic membrane.

### Medipeptin A Binds to Lipoteichoic Acid and Lipid II

To assess whether medipeptin A has LTA binding action, bacterial growth assays were performed in 96-well plate with or without LTA. The results showed that cells treated with medipeptin A at a concentration of 3X MIC value grew and untreated cells when both of them were exposed to LTA (Figure 4C), which demonstrates that medipeptin A has an LTA binding capacity. Furthermore, the addition of lipid II originating from Gram-positive bacteria (lipid II-Lys) decreased the inhibition zone of medipeptin A toward *S. aureus* (Figure 4D), suggesting that medipeptin A binds to Gram-positive-type lipid II as well. Moreover, medipeptin A still showed similar inhibition zone of *S. aureus* before and after heat treatment (100°C, 30min), suggesting that the activity of medipeptin A is heat-stable.

## Discussion

In this study, we isolated and characterized *Pseudomonas mediterranea* EDOX from healthy tomato leaf endosphere. This strain can produce medipeptins to exert strong inhibitory activity against plant and human pathogens. In addition, we sequenced the genome of strain EDOX and conducted *in silico* analysis of BGCs. Furthermore, two CLPs, designated as medipeptins, were purified and characterized and their encoding gene cluster was identified. Their predicted sequence is shown in Figure 3. Subsequently, we investigated the mechanisms of medipeptins against Gram-positive and Gram-negative pathogens. Medipeptin A, binding to both LTA and lipid II as well as forming pores in membranes, is bacteriocidal against Gram-positive bacteria (*Staphylococcus aureus* subsp. *aureus* 5334R4). In contrast, medipeptin A acts as a bacteriostatic antibiotic against Gram-negative pathogens (*Xanthomonas translucens* pv. *graminis* LMG587). These findings are suggesting that medipeptins have potential to make contribute to develop new antibiotics and provide alternatives treatments for pathogens. Interestingly, *P. mediterranea* is a pathogenic species for plants such as *P. mediterranea* CFBP 10906 (Alippi and López, 2010). However, our strain *P. mediterranea* EDOX was isolated from leave endosphere of a healthy tomato plant. It suggests that *P. mediterranea* EDOX may be a true endophyte and play a beneficial role in tomato plants. This phenomenon is reasonable since the different behavior of same species can be explained by rather subtle differences (e.g., the expression of genes involved in plant defense reactions; Sheibani-Tezerji et al., 2015) and the influences from the host and/or the environmental conditions (Brader et al., 2017). For instance, *P. syringae* is a well-known pathogen worldwide, while *P. syringae* 260-02 was shown to promote plant growth and exert biocontrol of *P. syringae* pv. tomato DC3000, *Botrytis cinerea*, and *Cymbidium Ringspot* (Passera et al., 2019). The biocontrol ability and its mechanisms of *P. mediterranea* EDOX need to be further research.

Genome mining has become a powerful tool for the discovery of microbial natural products and understanding the activity profiles of bacteria. One of the advantages of this approach is that it unveils the full biosynthetic capacity of a microorganism (Jahanshah et al., 2019). The ever-increasing availability of genome sequencing data and the bioinformatic annotation of biosynthetic gene clusters (BGCs) facilitates the discovery of novel natural products. The inspection of a new *Pseudomonas* genome allowed us to predict the peptide sequence of multiple new variants and to identify potential producers of CLPs.

The BGC for biosynthesis of medipeptins in *Pseudomonas* sp. EDOX is quite similar to that of some known CLPs BGCs in *Pseudomonas* strains, including corpeptin BGC and nunapeptin BGC (Michelsen et al., 2015; Strano et al., 2015), but the structure and function of the potential CLPs produced by strain EDOX have not yet been investigated. The biosynthesis of medipeptin A and B is catalyzed by three NRPS genes (*mepA*, *mepB*, and *mepC*) in a collinear manner (Figure 3). By combining genome mining and LC–MS/MS analysis, we have determined the molecular structure of medipeptin A and medipeptin B. Medipeptins were produced as a family of cyclic lipopeptides of 22 amino acid residues with molecular masses 2082.20 [M+H]^+^ (medipeptin A) and 2051.18 [M+H]^+^ (medipeptin B; Figure 1).

Even though there are some reports showing the antimicrobial and/or antifungal activity of similar CLPs (corpeptin, nunapeptin, etc.), the antimicrobial mode of action of them is still not clear. To this end, we investigated the antimicrobial mechanisms of medipeptin A. In this study, we show that the newly discovered medipeptin A exerts its potent antimicrobial activity against Gram-positive pathogens *via* binding to both LTA and lipid II and probably by subsequently forming pores in membranes. LTA is a crucial constituent of Gram-positive cell envelopes, consisting of a poly (GroP) backbone linked to a glycolipid membrane anchor (Edgar et al., 2019). LTA, which is anchored to the cell envelope, could control autolysin activity. The interaction of CLPs with LTAs can result in loss of regulation of autolysins, which then causes autolysis by hydrolyzing peptidoglycan chains and peptide bridges of murein (Bierbaum and Sahl, 1985). In addition, the interaction between LTA and CLPs is believed to facilitate penetration of CLPs through the thick peptidoglycan layer, allowing the CLPs to reach and act on the cytoplasmic membrane (Yasir et al., 2019). In our study (Figure 4C), the added LTAs affect the activity of medipeptin A against *S. aureus*, which suggests that medipeptin A binds to LTA. Since lipid II is the bactoprenol-bound precursor of the bacterial cell wall, binding to lipid II could block the incorporation of lipid II into peptidoglycan, resulting in slow cell lysis and/or enhances the stability of pore formation, which increases the activity of the peptide (Bauer and Dicks, 2005). Based on the lipid II binding assay and the fact that the antimicrobial activity of medipeptin A decreased after treatment of the cells with lipid II (Figure 4D), we suggest that medipeptin A can also bind to lipid II to exert its activity against *S. aureus*. In addition, we observed that cells of *S. aureus* could be stained red after treated with medipeptin A, indicating that medipeptin A shows pore-forming activity in biological membranes of *S. aureus* (Figure 4B).

To conclude, we isolated *P. mediterranea* EDOX from tomato endosphere. One BGC encoding the novel medipeptin biosynthesis cluster was identified from the genome sequence of EDOX. The purified medipeptin A has bactericidal activity against Gram-positive pathogens, but a bacteriostatic activity toward Gram-negative pathogens. We also show that medipeptin A combats Gram-positive pathogens through binding both to LTA and lipid II, as well as by forming pores in membranes. This study provides novel insights into the antimicrobial mode of action of medipeptin A, which broadens the array of candidates for drug development and biocontrol agents.

## Data Availability Statement

The original contributions presented in the study are included in the article/supplementary material, further inquiries can be directed to the corresponding author.

## Author Contributions

LZ and OK conceived the study, designed the experiments, and corrected the manuscript. AJ and YY assembled the draft genome. LZ performed the experiments and wrote the draft manuscript. All authors contributed to the article and approved the submitted version.

## Funding

LZ was financially supported by the China Scholarship Council (201606910037). YY was financially supported by the China Scholarship Council (201904910477).

## Conflict of Interest

The authors declare that the research was conducted in the absence of any commercial or financial relationships that could be construed as a potential conflict of interest.

## Publisher’s Note

All claims expressed in this article are solely those of the authors and do not necessarily represent those of their affiliated organizations, or those of the publisher, the editors and the reviewers. Any product that may be evaluated in this article, or claim that may be made by its manufacturer, is not guaranteed or endorsed by the publisher.

## Footnotes

^1^ http://combo.dbe.unifi.it/medusa
